# Development of Multiple Real-Time Fluorescent Quantitative PCR for *Vibrio* Pathogen Detection in Aquaculture

**DOI:** 10.3390/vetsci12040327

**Published:** 2025-04-02

**Authors:** Binzhe Zhang, Yulie Qiu, Chenxi Shi, Jian Zhang

**Affiliations:** 1 School of Ocean, Yantai University, Yantai 264005, China; 2 Shandong Engineering Research Center of Healthy Land-Sea Relay Farming of Economic Fish, Yantai 264005, China; 3 Yantai Engineering Research Center of Deep-Sea Aquaculture of Economic Fish, Yantai 264005, China

**Keywords:** *Vibrio*, TaqMan probe, multiplex real-time PCR, differential diagnosis

## Abstract

The *Vibrio* genus represents a critical group of bacterial pathogens in the marine environment globally, leading to massive mortality in the aquaculture industry. Diagnosing vibriosis, an infection caused by *Vibrio* species, in clinical samples poses challenges due to its non-specific clinical manifestations. In this study, we developed a TaqMan probe-based multiplex real-time PCR method for the simultaneous detection and quantification of four *Vibrio* pathogens: *Vibrio anguillarum* (Va), *Vibrio alginolyticus* (Val), *Vibrio harveyi* (Vh), and *Vibrio scophthalmi* (Vsc). The assay targets conserved intra-species regions and specific inter-species regions using specific primers and TaqMan probes to ensure specificity. Sensitivity analysis demonstrated that the multiplex real-time PCR assay could simultaneously detect the four different bacteria, with detection limits of 26–60 copies per reaction, making it 100 times more sensitive than conventional PCR assays. Additionally, the assay exhibited high reproducibility, with intra- and inter-group coefficients of variation below 1.4%. A total of 63 clinical samples was analyzed using this established assay, which successfully detected both single and mixed infections. These results demonstrate that the multiplex quantitative PCR assay is a rapid, specific, and sensitive diagnostic tool for the detection of Va, Val, Vh, and Vsc, making it suitable for monitoring these bacteria in both single- and co-infected clinical samples.

## Simple Summary

Vibriosis, caused by various *Vibrio* species, significantly impacts marine biomass and leads to considerable economic losses in the aquaculture industry. Notably, *Vibrio anguillarum*, *Vibrio alginolyticus*, *Vibrio harveyi*, and *Vibrio scophthalmi* are major bacterial pathogens affecting the marine aquaculture sector. Thus, this study aimed to develop a rapid, accurate, and sensitive multiplex diagnostic method for the early detection of these four *Vibrio* species. A TaqMan probe-based multiplex real-time PCR method was developed, exhibiting four key characteristics: (1) high sensitivity, with detection limits 100 times more sensitive than conventional PCR assays; (2) high specificity, accurately and specifically detecting *V. anguillarum*, *V. alginolyticus*, *V. harveyi*, and *V. scophthalmi*; (3) multiplex detection, enabling the simultaneous detection of *V. anguillarum*, *V. alginolyticus*, *V. harveyi*, and *V. scophthalmi* in a single reaction; and (4) time efficiency, with detection results obtainable within one hour. These advancements facilitate the early detection and monitoring of these bacterial pathogens in both single and co-infected samples.

## 1. Introduction

With the ongoing development of the mariculture industry, a diverse array of pathogens continues to emerge, leading to significant economic losses. Among these, bacterial infections are the most prevalent in aquaculture, characterized by their diversity and global distribution, with the potential to infect humans and other mammals [1,2]. The genus *Vibrio* is a major pathogenic microorganism, widely present in marine and freshwater ecosystems. Outbreaks of vibriosis have a severe impact on marine biomass and result in substantial economic losses in the aquaculture industry [3]. Early diagnosis is essential for the prevention and effective treatment of vibriosis; therefore, a rapid, accurate, multiplex, and sensitive diagnostic method is imperative.

*Vibrio anguillarum*, *Vibrio alginolyticus*, *Vibrio harveyi*, and *Vibrio scophthalmi* are significant bacterial pathogens affecting marine fish. *Vibrio anguillarum* is a halophilic pathogenic bacterium that is widely distributed in coastal and estuarine seawater [4]. It serves as a representative opportunistic bacterial pathogen for marine fish, shellfish, mollusks, and crustaceans, contributing to substantial economic losses in aquaculture [5]. This bacterium is capable of infecting at least 50 fish species, including Atlantic salmon (*Salmo salar*), rainbow trout (*Oncorhynchus mykiss*), turbot (*Scophthalmus maximus*), black sea bream (*Sparus macrocephalus*) and Japanese flounder (*Parallichthys olivaceus*) [6,7]. *V. alginolyticus*, initially classified as *V. parahaemolyticus*, is another halophilic bacterium predominantly found in marine and estuarine environments. It is regarded as one of the most detrimental *Vibrio* species to aquatic animals [8,9]. Systemic infections caused by *V. alginolyticus* have been documented in numerous fish and shrimp species, such as alfonsino (*Sparus aurata*), cobia (*Rachycentron canadum*), giant river prawn (*Macrobrachium rosenbergii*, *Epinephelus malabaricus*), South America white shrimp (*Penaeus vannamei*), and Japanese prawn (*Penaeus japonicas*), resulting in significant economic losses in aquaculture globally [10,11,12,13,14,15]. *V. harveyi* is recognized as a significant pathogen responsible for considerable mortality rates among various wild and cultured fish and invertebrates across a broad geographical range [16]. To date, *V. harveyi* has been isolated from a diverse array of marine fish and shrimp species worldwide, including Atlantic flounder (*Paralichthys dentatus*), *S. salar*, *O. mykiss*, silver perch (*Lates calcarifer*), orange-spotted grouper (*Epinephelus coioides*), sugpo prawn (*Penaeus monodon*) [17], and *P. vannamei* [17,18,19,20]. *V. scophthalmi* is an opportunistic pathogen of aquatic animals which was first isolated from the intestine of turbot (*S. maximus*) in 1997 [21]. Fish subjected to stressful conditions exhibit increased susceptibility to this bacterial strain [22]. To date, *V. scophthalmi* has also been isolated from other fish species, such as *P. olivaceus*, Japanese eel (*Anguilla japonica*), manila clam (*Ruditapes philippinarum*), and bluefin tuna (*Thunnus maccoyii*), resulting in mortality rates ranging from 30% to 90% among infected fish [22,23,24,25].

Due to the significant economic impact of these pathogens, the development of rapid and reliable detection methods is essential for preventing their further dissemination and facilitating appropriate therapeutic interventions. *Vibrio* species exhibit numerous similarities within this genus, including morphology, virulence factors, genome sequences, and clinical symptoms, which complicate the specific differentiation of one *Vibrio* species from another [26,27]. Current detection methodologies primarily rely on traditional culture-based techniques, as well as immunological and molecular biological detection technologies. While conventional symptom-based or culture-based methods can identify *Vibrio* infections, they often lack the specificity required to accurately distinguish between different *Vibrio* species [28,29]. PCR-based molecular diagnostics are widely employed in clinical settings due to their high specificity [30]. Numerous conventional PCR-based methods have been developed for the detection of *V. anguillarum*, *V. alginolyticus*, *V. harveyi*, and *V. scophthalmi*. However, these methods tend to be labor intensive and less sensitive [31,32,33,34]. Moreover, isothermal amplification techniques and real-time PCR have also been developed for the detection of certain bacterial pathogens [34,35,36,37,38,39]. Although these methods offer increased specificity, sensitivity, and time efficiency, they do not fully meet the clinical requirements for the detection of multiple pathogens.

TaqMan probe-based qPCR offers a rapid, specific, sensitive, and reproducible approach for pathogen detection and quantification, while multiplex PCR provides the advantages of high throughput and efficiency. Although the qPCR method is more expensive than conventional PCR, culturing methods, and the immune colloidal gold technique, it offers the advantage of detecting bacterial pathogens at the early stages of disease, thereby facilitating early prevention and treatment. TaqMan probe-based multiplex real-time PCR (multiplex qPCR) integrates multi-PCR and TaqMan probe-based qPCR technologies, offering advantages such as high sensitivity, the simultaneous detection of multiple targets, time efficiency, and reagent conservation [40]. In this study, a TaqMan probe-based multiplex real-time PCR assay was developed using the *empA*, *toxR*, *vhhP2*, and *luxR* genes as target markers to simultaneously detect *V. anguillarum*, *V. alginolyticus*, *V. harveyi*, and *V. scophthalmi*. This method demonstrates high specificity and sensitivity, is time-efficient, and possesses strong quantitative capabilities, making it a promising tool for the early diagnosis of infections caused by multiple pathogens.

## 2. Materials and Methods

### 2.1. Ethics Statement

Live animal experiments were performed in accordance with the guidelines of “Regulations for the Administration of Affairs Concerning Experimental Animals” promulgated by Shandong Province. Experiments involving live animals were approved by the Ethics Committee of Yantai University, with the ethical approval code No. 20230503.

### 2.2. Bacteria, Clinical, and Environmental Samples

Bacterial strains *Edwardsiella piscicida* (Ep), *Photobacterium damselae* (Pd), *Pseudomonas fluorescens* (Pf), *Vibrio anguillarum* (Va), and *Vibrio harveyi* (Vh) have been described previously [41,42,43,44]. Strains *Vibrio alginolyticus* (Val), *Vibrio rotiferianus* (Vro), *Leclercia adecarboxylata* (La), *Vibrio hyugaensis* (Vhy), *Haemophilus piscium* (Hp), *Vibrio azureus* (Vaz), and *Vibrio scophthalmi* (Vsc) were isolated and stored in our lab. All bacterial strains were cultured in Luria–Bertani broth (LB) medium at 28 °C for 12 h with constant shaking at 180 rpm under aerobic conditions with a rotary shaking (Zhichu, Shanghai, China). All bacterial species were further confirmed via 16S rDNA gene sequencing, conducted by Sangon Biotech Co., Ltd. (Shanghai, China).

For the preparation of clinical samples, 63 fish tissue samples were obtained. Briefly, 45 clinically healthy black rockfish (*Sebastes schlegeli*) with an average weight of 24.5 g were purchased from a commercial fish farm in Yantai, Shandong Province. The fish were maintained in aerated tanks (9 fish/tank) containing freshly prepared artificial seawater (changed daily) with a salinity of 30‰, a pH of 8.5, and a temperature of 20 °C. The bacterial strains Va, Val, Vh, and Vsc were cultured in LB medium as described above, and the turbidity of each strain was measured in a spectrophotometer (ThermoFisher, Waltham, MA, USA). When the optical density at 600 nm (OD_600_) reached 0.8–1.0, the bacterial cells were harvested through centrifugation at 5000× *g* for 2 min. The bacterial pellet was then washed 3 times with sterile phosphate-buffered saline (PBS) and adjusted to a concentration of 10^7^ cfu/mL with PBS. The fish were divided into five groups and subjected to intraperitoneal injection with Va, Val, Vh, Vsc, or PBS, respectively. At 12, 24, and 48 h post-challenge (hpc), the fish (3 per time point) were euthanized using an overdose of tricaine methanesulfonate (Sigma, St. Louis, MO, USA). Then, the surfaces of the fish were disinfected with ethanol, and the liver, spleen, and kidney were collected under sterile conditions. For each fish, the three tissues were pooled, homogenized in a ten-fold volume of PBS, and subjected to bacterial examination via plate counts, as previously described [45]. Subsequently, positive samples containing different bacterial pathogens were selected and combined to create double-positive, triple-positive and quadruple-positive samples.

For the preparation of environmental samples, 21 sea-water and 21 sediment samples were collected from the intertidal zone of Yantai, China. Prior to experimentation, these samples were assessed for the presence of Va, Val, Vh, and Vsc using bacterial enrichment culture and PCR analysis. Subsequently, varying concentrations of Va, Val, Vh, and Vsc were added, and were verified as described above. A total of 42 positive samples (comprising 15 sea-water and 15 sediment samples) and 12 negative samples (comprising 6 sea-water and 6 sediment samples) were prepared for further qPCR analysis.

### 2.3. DNA Extraction

For the isolation of bacterial DNA, the bacterial strains were cultured in LB medium to an OD_600_ = 1.5. Subsequently, 1 mL bacterial suspension from each strain was collected and centrifuged at 5000× *g* for 2 min, and the bacterial pellet was subjected to DNA extraction using the TIANamp Bacteria DNA Kit (Tiangen Biotech Co, Ltd., Beijing, China) in accordance with the manufacturer’s instructions. For clinical samples, 200 μL of tissue homogenate was utilized for DNA extraction employing the CTAB method [46]. The extracted DNA was dissolved in 100 μL of nuclease-free water and stored at −20 °C for subsequent analyses.

### 2.4. Standard Recombinant Plasmids Construction

The *empA* gene (GenBank accession no. L02528), *toxR* gene (KJ579443), *vhhP2* gene (FJ025787), and *luxR* gene (JN684210) were selected as target genes for the detection of Va, Val, Vh, and Vsc, respectively. Partial sequences of the *empA*, *toxR*, *vhhP2,* and *luxR* genes were amplified using the primer pairs empA-F/R, toxR-F/R, vhhP2-F/R, and luxR-F/R (Table 1), respectively, and subsequently inserted into the pMD-19^T^ vector (TAKARA, Dalian, China). The recombinant plasmids were verified through sequencing in Sangon Biotech Co., Ltd., and were designated as pVa, pVal, pVh, and pVsc, respectively. The concentration of the recombinant plasmids was measured using a Nano-500 micro-spectrophotometer (Allsheng, Hangzhou, China). The DNA copy numbers were calculated using the following formula: DNA copy number (copies/μL) = [6.02 × 10^23^ × plasmid concentration (ng/μL) × 10^−9^]/[DNA length × 660].

The standard recombinant plasmids were serially diluted (10-fold) ranging from 1 × 10^8^ to 1 × 10^0^ copies/μL and stored at −20 °C for further use.

### 2.5. TaqMan Real-Time Fluorescent Quantitative PCR (qPCR) Primer Design

For the analysis of intraspecific conserved sequences and interspecific specific sequences, multiple sequence alignment was conducted using gene sequences obtained from the National Center for Biotechnology Information (NCBI) database, employing DNAMAN software (v7.0) (Table S1). Primers (empA-qF/qR, toxR-qF/qR, vhhP2-qF/qR, and luxR-qF/qR) and TaqMan fluorescence probes (empA-P, toxR-P, vhhP2-P, and luxR-P) were designed for qPCR (Table 1). Different fluorescence labels, FAM (6-carboxyfluorescein), HEX (6-carboxy-4′,5′-dichloro-2′,7′-dimethoxyfluorescein ester dye), ROX (Carboxy-X-Rhodamine), and CY5 (Cy Dye 5), were incorporated into the probes to distinguish different bacteria. Specificity of the primers and probes was confirmed through a Primer-BLAST search against the NCBI database. Additionally, OligoEvaluator software “http://www.oligoevaluator.com (accessed on 13 September 2023)” was utilized to assess the potential formation of primer dimers and hairpins. Finally, the synthesis and purification of the primers and probes were carried out by Sangon Biotech Co., Ltd., employing high-performance liquid chromatography techniques.

### 2.6. Optimization of Reaction Conditions for Multiplex qPCR

The reaction system and conditions for the multiplex qPCR were optimized by referencing the singleplex assay, utilizing the Pro Taq HS Premix Probe real-time PCR Kit III (Accurate Biology, Changsha, China), and conducted on the Bio-Rad CFX96 platform (Bio-Rad, Hercules, CA, USA). A series of ten concentration gradients for both forward and reverse primers (ranging 0.1 μmol/L~1 μmol/L, with increments of 0.1 μmol/L), and eight concentration gradients of probe (concentration range: 0.1 μmol/L~0.8 μmol/L; concentration gradient difference: 0.1 μmol/L) were set to determine the optimal primer and probe concentrations. The optimal annealing/extension temperature was evaluated across three temperatures (58 °C, 60 °C, and 62 °C). The temperature of optimal primers and probes, as well as the annealing/extension temperature, was selected based on amplification efficiency, determined from the Cq (cycle of quantification) values and the fluorescence intensity.

### 2.7. Establishment of Standard Curves of the qPCR

To construct standard curves, each standard recombinant plasmid was subjected to a 10-fold serial dilution, resulting in 7 dilution gradients ranging from 1 × 10^9^ to 1 × 10^4^ copies/μL. qPCR was conducted using these diluted plasmids in accordance with the optimized reaction system and procedures. The standard curve for qPCR was generated by plotting the logarithmic value of the plasmid copy number on the *x*-axis against the corresponding Cq value on the *y*-axis.

### 2.8. Specificity Test

To avoid false positives caused by other bacteria that could be present in this multiplex qPCR assay, the DNA templates extracted from other 8 bacterial strains, i.e., Ep, Pd, Vro, Pf, La, Vhy, Hp, and Vaz, were amplified, and ddH_2_O was used as a negative control. The specificity tests were performed under optimal conditions and system and repeated more than thrice. The templates were verified via 16S rRNA gene amplification and sequencing.

### 2.9. Sensitivity Test

False negatives are typically identified using the detection limit. Herein, the standard plasmids, pVa, pVal, pVh, and pVsc, were subjected to a 10-fold dilution to achieve a final concentration ranging from 10^5^ to 10^0^ copies/µL in nuclease-free water, in order to evaluate the sensitivity of the established multiplex qPCR. Optimal reaction conditions were used for multiplex qPCR amplification, with diluted standard plasmids serving as templates, and ddH_2_O as a negative control. Each concentration or negative control was analyzed in triplicate. For the conventional PCR analysis, diluted standard plasmids pVa, pVal, pVh, and pVsc were amplified using the primer pairs empA-F/R, toxR-F/R, vhhP2-F/R, and luxR-F/R, respectively. The conventional PCR was performed on a Bio-rad MyCycler PCR Thermal Cycler System (Bio-Rad, Hercules, WA, USA), and the reaction conditions were as follows: an initial denaturation at 95 °C for 3 min, followed by 35 cycles of denaturation at 95 °C for 30 s, annealing at 55 °C for 30 s, an extension at 72 °C for 90 s, and a final extension at 72 °C for 10 min. For agarose gel electrophoresis, a gel was prepared using 1 × TAE buffer (Solarbio, Beijing, China) with 1.5% agarose (Solarbio). The D2000 DNA Ladder (Solarbio) was employed as a standard marker to estimate the sizes of the amplified products.

### 2.10. Repeatability Test

The standard plasmids, pVa, pVal, pVh, and pVsc, were subjected to a 10-fold serial dilution to achieve final concentrations ranging from 10^6^ to 10^4^ copies/µL in nuclease-free water. This was conducted to evaluate the repeatability and reproducibility of the established multiplex qPCR. For multiplex real-time qPCR amplification, diluted standard plasmids were employed as templates. For intra-group repeatability analysis, qPCR was performed in triplicate for each concentration gradient. For inter-group repeatability analysis, each template was analyzed thrice with different participants every other week under the same conditions. The repeatability and reproducibility of the method were subsequently assessed using the coefficient of variation (CV), where a smaller CV value indicates greater data stability. The more stable the data, the smaller the CV value. The CV values were determined using the following formula: CV = [Standard Deviation]/[Mean] × 100.

### 2.11. Clinical Sample Testing

To evaluate the applicability of the multiplex qPCR assay for Va, Val, Vh, and Vsc detection in clinical and environmental samples, total DNA was extracted from the 105 samples prepared above. Among the clinical samples, 9 were negative control samples, 24 were single-positive samples, 18 were double-positive samples, 9 were triple-positive samples, and 3 were quadruple-positive samples. Among the sea-water samples, 6 were negative control samples, 12 were single-positive samples, and 3 were quadruple-positive samples. Among the sediment samples, 6 were negative control samples, 12 were single-positive samples, and 3 were quadruple-positive samples. Subsequently, the multiple qPCR assay was conducted on all 105 samples under the optimal reaction system and reaction conditions, and the coincidence rate between the detection results and the expected results was calculated. During these experiments, the standard plasmids, pVa, pVal, pVh, and pVsc, were used as positive controls.

### 2.12. Statistical Analysis

Standard curves, correlation coefficient (R^2^) values, and amplification efficiency (E) were analyzed using software supplied by the ABI PRISM 7500. The repeatability analysis was conducted using Microsoft Excel 2013 (Microsoft, Redmond, WA, USA).

## 3. Results

### 3.1. Primers and Probe Designed for qPCR Assay

**Figure 1:**
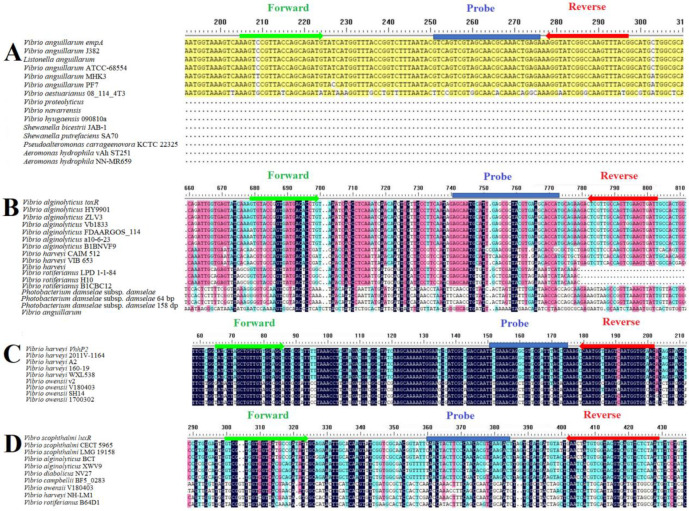
Multiple sequence alignment of target genes with their homologous sequences. (**A**) *empA* gene; (**B**) *toxR* gene; (**C**) *vhhP2* gene; (**D**) *luxR* gene. Black indicates the consensus residues; pink indicates the residues that are ≥75% identical among the aligned sequences; blue indicates the residues that are ≥50% and <75% identical among the aligned sequences; and yellow indicates residues that are ≥25% and <50% identical among the aligned sequences. The red/green arrows and blue horizontal lines indicate primer and probe sequences, respectively.

The genes *empA*, *toxR*, *vhhP2,* and *luxR* were selected for the detection of Va, Val, Vh, and Vsc, respectively. Sequences of the *empA*, *toxR*, *vhhP2,* and *luxR* genes were retrieved from GenBank for multiple sequence alignment using DNAMAIN. To develop specific primers and probes, highly conserved intraspecific regions and specific interspecific regions were identified, and primers were designed utilizing AllelelD 6.0 software (Figure 1 and Table 1).

### 3.2. Optimization of qPCR Reaction Conditions

**Figure 2:**
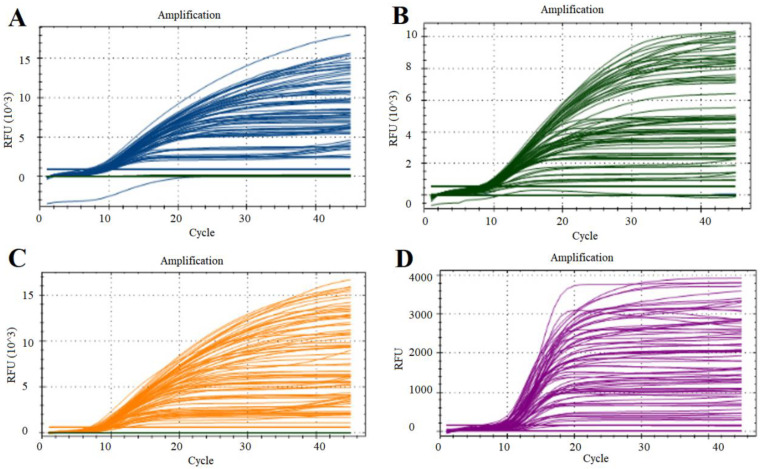
Optimization of the single qPCR conditions. Amplification curves based on the qPCR amplification of plasmids pempA (**A**), ptoxR (**B**), pvhhP2 (**C**), and pluxR (**D**). RFU: relative fluorescence unit.

Through the optimization of primer and probe concentrations via single qPCR, the optimal working concentrations of primers for Va, Val, Vh, and Vsc were established at 0.3, 0.2, 0.4, and 0.2 μmol, respectively, while the optimal concentrations for the probes were 0.5, 0.6, 0.7, and 0.3 μmol, respectively (Figure 2 and Table 2). The reaction mixture comprised 10 μL of 2 × universal probe mix (Accurate Biology, Beijing, China), the specified primers and probes, 1 μL of each standard recombinant plasmid, and nuclease-free water to a total volume of 20 μL (Table 2). Variations in annealing temperatures did not significantly affect the results, leading to the selection of 60 °C as the standard annealing temperature. The final optimized reaction program was conducted as follows: pre-denaturation at 95 °C for 5 min, followed by 40 cycles at 95 °C for 15 s, and 60 °C for 30 s. The fluorescence channels for multiple qPCR analysis were set as follows: channel 1, FAM; channel 2, HEX; channel 3, ROX; and channel 4, Cy5.

### 3.3. Standard Plasmid Construction and Standard Curve Establishment

For the construction of standard plasmids, partial sequences of the *empA* gene (800 bp), *toxR* gene (734 bp), *vhhP2* gene (588 bp), and *luxR* gene (615 bp) were amplified and subsequently inserted into the pMD-19T vector (Figure S1). The resultant positive recombinant plasmids were extracted, sequenced, and designated as pVa, pVal, pVh, and pVsc. The purity and concentration of these plasmids were assessed using an Ultramicro spectrophotometer Nano 300 (Allsheng, Hangzhou, China). The copy numbers of pVa, pVal, pVh, and pVsc were determined to be 4.03 × 10^10^, 3.35 × 10^10^, 6.01 × 10^10^, and 2.59 × 10^10^ copies/μL, respectively.

**Figure 3:**
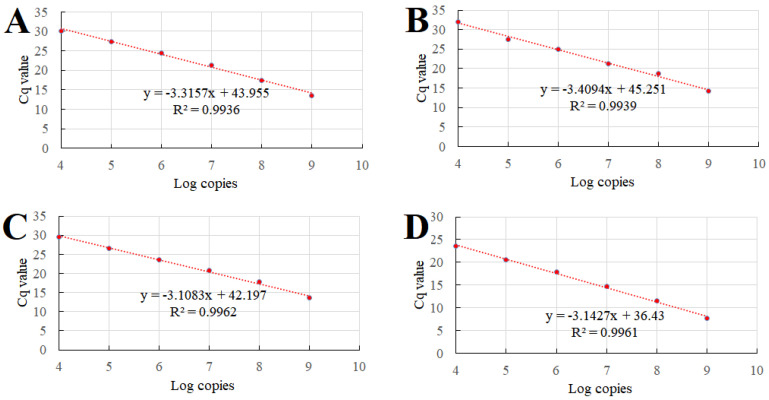
Standard curves of the multiplex qPCR method. Ten-fold serially diluted plasmids (from 10^9^ to 10^4^ copies/µL) were amplified with different primer/probe sets targeting Va (**A**), Val (**B**), Vh (**C**), and Vsc (**D**). The Cq values were then plotted against the logarithmic plasmid copy number. R^2^ = correlation coefficient.

For the establishment of standard curves, the standard plasmids were serially diluted in a 10-fold gradient ranging from 10^9^ to 10^4^ copies/µL and subsequently amplified via quantitative PCR (qPCR) under optimized reaction conditions. Four standard curves were generated by plotting the obtained quantification cycle (Cq) values on the *y*-axis against the logarithm of the plasmid concentration on the *x*-axis. The optimal standard formula, R^2^, and E values were as follows: Va, y = −3.3157x + 43.955, R^2^ = 0.9963, E = 1.00; Val, y = −3.4094x + 45.251, R^2^ = 0.9939, E = 0.96; Vh, y = −3.1083x + 42.197, R^2^ = 0.9962, E = 1.09; Vsc, y = −3.1427x + 36.43, R^2^ = 0.9961, E = 1.08 (Figure 3). The established curves exhibit excellent correlation coefficients, indicating that the TaqMan probe-based multiplex qPCR method developed is both reliable and valid.

### 3.4. Specificity of the Multiplex qPCR

**Figure 4:**
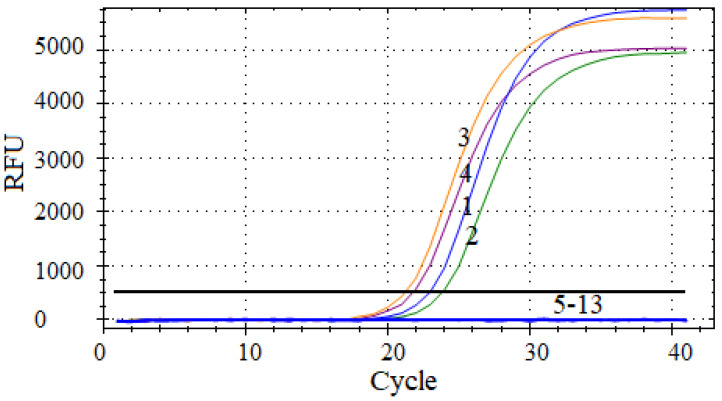
Specificity analysis of the multiplex qPCR method. Genomic DNA of (1) Va, (2) Val, (3) Vh, (4) Vsc, (5) Ep, (6) Pd, (7) Vro, (8) Pf, (9) La, (10) Vhy, (11) Hp, (12) Vaz, and (13) negative control amplified by the multiplex qPCR.

The multiplex qPCR assay was conducted to detect various bacterial DNA sequences under uniform optimal reaction conditions. The results demonstrate that only DNA isolated from Va, Val, Vh, and Vsc was successfully detected in its specific fluorescence channel. Importantly, the DNA from other bacterial species, including Ep, Pd, Vro, Pf, La, Vhy, Hp, and Vaz, was not amplified, underscoring the high specificity of the developed multiplex qPCR method (Figure 4).

### 3.5. Sensitivity of the Multiplex qPCR

**Figure 5:**
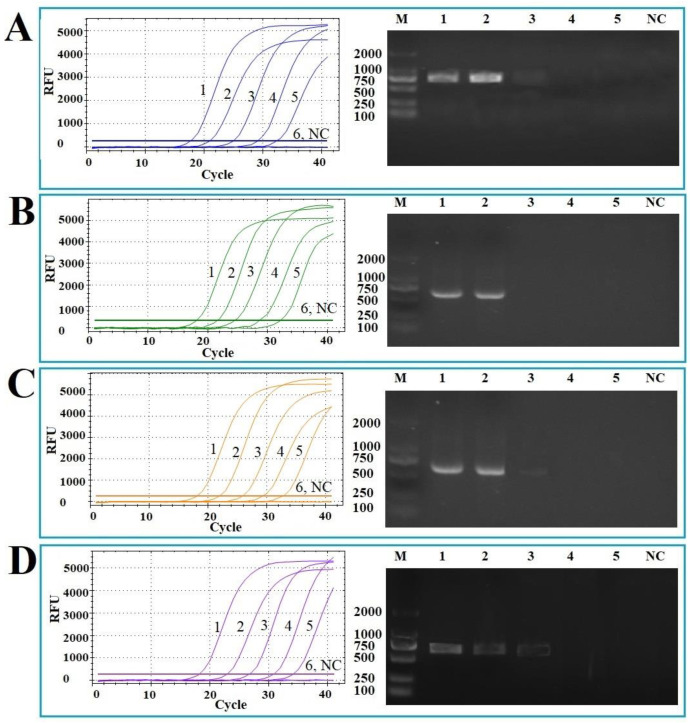
Sensitivity analysis of the multiplex qPCR method. Ten-fold serially diluted plasmids (from 10^5^ to 10^0^ copies/µL) were amplified with different primer/probe sets targeting Va (**A**), Val (**B**), Vh (**C**), and Vsc (**D**), and the multiplex qPCR results were compared with the results of conventional PCR. 1–6: 10^5^–10^0^ copies/µL standard plasmids; NC: negative control.

For sensitivity analysis, six concentrations (10^5^ copies/μL–10^0^ copies/μL) of different standard plasmids were evaluated and compared using both the qPCR and conventional PCR techniques. The detection limits of the qPCR for Va, Val, Vh, and Vsc were determined to be 4.0 × 10^1^, 3.4 × 10^1^, 6.0 × 10^1^, and 2.6 × 10^1^ copies/uL, respectively. In contrast, the detection limits for conventional PCR were 4.0 × 10^3^, 3.4 × 10^3^, 6.0 × 10^3^, and 2.6 × 10^3^ copies/uL, respectively. The average Cq values for all standard plasmids at 10^1^ copies/uL were greater than 35, and thus Cq values ≥ 35 were defined as the critical point of negative. Compared with the traditional PCR, the sensitivity of the developed qPCR increased by 100 times (Figure 5).

### 3.6. Repeatability of the Multiplex qPCR

The repeatability and reproducibility of the developed multiplex quantitative PCR (qPCR) assay were assessed utilizing four standard plasmids, diluted ten-fold, ranging from 10^6^ to 10^4^ copies/µL, as templates. The coefficients of variation (%CV) were employed as the metric for evaluation. The findings revealed that the intra-assay and inter-assay CVs ranged from 0.20% to 1.39% and from 0.20% to 1.26%, respectively (Table 3), demonstrating the assay’s excellent repeatability and high accuracy.

### 3.7. Testing of Clinical and Environmental Samples Using the Multiplex qPCR

The applicability of the multiplex qPCR was evaluated using 63 clinical samples and 42 environmental samples. In clinical samples, the assay identified 14.29% as negative, 38.10% as single-positive, 28.57% as double-positive, 14.29% as triple-positive, and 4.76% as quadruple-positive (Table 4 and Table S2). In environmental samples, the assay identified 28.57% as negative, 57.14% as single-positive, and 14.29% as quadruple-positive (Table 4 and Table S3). The detection method exhibited an accuracy rate of 100%, underscoring the robust accuracy of the established multiplex qPCR method in the detection of clinical samples.

## 4. Discussion

Vibriosis, caused by *Vibrio* species, represents one of the most prevalent diseases in marine aquaculture, posing a significant threat to both wild and farmed marine species globally [47]. The rapid expansion of the aquaculture industry has facilitated numerous outbreaks and the spread of various *Vibrio* species, especially Va, Val, Vh, and Vhc, resulting in substantial economic losses in mariculture worldwide [4,8,16,22]. These bacterial pathogens exhibit a high likelihood of co-infection and cross-species transmission, and share similar clinical symptoms, complicating accurate diagnosis [3,48]. Consequently, the development of effective and specific diagnostic tools is essential for the detection of these bacteria. Over recent years, molecular diagnostic methods have undergone significant advancements. Currently, several traditional PCR, quantitative PCR (qPCR), multiplex PCR, and isothermal amplification techniques have been developed for the detection of Va, Val, Vh, or Vsc [34,49,50,51,52]. Nonetheless, no existing method can simultaneously detect all four bacteria in a single reaction.

TaqMan probe-based qPCR is widely regarded as the most reliable method for detecting pathogens in humans, livestock, and aquatic organisms [53]. In this study, we integrated the benefits of both quantitative PCR (qPCR) and multiplex PCR for the detection of *Vibrio* species Va, Val, Vh, and Vsc, selecting the genes *empA*, *toxR*, *vhhP2*, and *luxR* as the respective target genes. The *empA* gene, which encodes a zinc metalloprotease, is recognized as a crucial virulence factor of the fish pathogen *V. anguillarum* [54]. The *empA* gene holds potential for bacterial species identification; to date, PCR and Loop-mediated Isothermal Amplification (LAMP) methods have been developed for the specific detection of *V. anguillarum* using *empA* as a target gene [33,55]. For the detection of *V. alginolyticus*, rapid detection methods such as LAMP and Recombinase-Aided Amplification (RAA) have been developed, targeting the virulence gene *toxR* [39,56,57]. The *toxR* gene is a transcriptional regulatory gene which is exhibited in all pathogenic strains of *V. alginolyticus* and plays a significant role in regulating the transcription of virulence genes [58]. The *toxR* gene exhibits high interspecific conservation and intraspecific hypervariability, making it a reliable molecular target for the identification of *Vibrio* species [39,56,57,59]. VhhP2 is an outer membrane protein ubiquitously found in the pathogenic strains of *V. harveyi*, but it is absent in most non-*V. harveyi* species. This characteristic makes *vhhP2* a specific marker for the precise identification of *V. harveyi* [53,60]. Several PCR-based methods have been developed utilizing the conserved sequence of *vhhP2* for the rapid and specific detection of *V. harveyi* [60,61]. *LuxR* serves as a master quorum-sensing regulator, influencing biofilm formation in *V. scophthalmi* and certain pathogenic *Vibrio* species [62]. The *luxR* gene of *V. scophthalmi* exhibits relatively low similarity with those of other bacterial species, making it a suitable target gene for *V. scophthalmi* detection using a LAMP method [63,64]. In this study, multiple sequence alignments were conducted, and both intraspecific conserved and interspecific variant regions of these genes were utilized to design species-specific primers and probes. Specificity analysis demonstrated that the multiplex qPCR method exhibited no cross-reactivity with each other or with eight other pathogens, including Ep, Pd, Vro, Pf, La, Vhy, Hp, and Vaz, thereby indicating high specificity.

In this study, a TaqMan probe-based multiplex quantitative PCR (qPCR) method was developed to enable the simultaneous detection of Va, Val, Vh, and Vsc within a single reaction system. The minimum detection limits for the multiplex qPCR were determined to be 4.0 × 10^1^, 3.4 × 10^1^, 6.0 × 10^1^, and 2.6 × 10^1^ copies/uL for Va, Val, Vh, and Vsc, respectively. This represents an enhancement in detection sensitivity ranging from 10- to 100-fold compared to traditional PCR methods [31,32,33,34]. Unlike single qPCR assays, multiplex qPCR is performed within the same reaction mixture, leading to more rapid substrate consumption and the generation of internal competition, which can result in a slight reduction in sensitivity. The detection limits achieved with the developed multiplex qPCR were comparable to or marginally higher than those reported for single qPCR assays in previous studies [34,36,37,65]. Similar findings were observed in a multiplex qPCR method designed for the detection of four feline diarrhea-associated viruses, suggesting that competition among primers, probes, and templates of different pathogens may reduce the sensitivity of multiplex detection methods [66].

The standard curves for Va, Val, Vh, and Vsc exhibit strong linearity and correlation, with the coefficient of determination (R^2^) exceeding 0.99, indicative of extremely high correlations. According to the MIQE guidelines [67], optimal amplification efficiencies should range between 90% and 110%. In this study, the amplification efficiency of the standard plasmids ranged from 96% to 108%, demonstrating high amplification efficiencies. Furthermore, the coefficients of variation (CVs) for both inter-assay and intra-assay replicates were below 1.4%, significantly lower than those reported for other multiplex qPCR methods [68,69,70], suggesting that the established qPCR method possesses high repeatability and stability.

A notable advantage of the multiplex qPCR is its capability to detect mixed target bacteria in complex clinical and environmental samples. To evaluate this advantage, 63 distinct artificial clinical samples and 42 environmental samples were prepared, encompassing single-, double-, triple-, and quadruple-infected samples. The established multiplex qPCR method successfully detected and differentiated the mixed bacteria in all 63 clinical samples and 42 environmental samples, demonstrating high accuracy. These findings of this study demonstrate that multiplex quantitative PCR (qPCR) methods are effective for the detection of Va, Val, Vh, and Vsc infections in fish or their existence in water and sedimental samples.

## 5. Conclusions

In this study, we have successfully developed, for the first time, a TaqMan multiplex qPCR technique capable of simultaneously and accurately detecting these pathogens. This method is characterized by high specificity and sensitivity, as well as rapid processing capabilities, making it an efficient tool for identifying bacterial pathogens with a substantial detection throughput. Furthermore, the method is cost-effective and time-efficient, rendering it particularly suitable for the detection of mixed pathogen infections in mariculture. This advancement facilitates early and rapid clinical detection and treatment in both clinical and field settings.

## Figures and Tables

**Table 1:** Primers and probes used in this study.

Target	Gene	Primer/Probe	Sequence (5′-3′)
Va	*empA*	empA-F	TTATATTGATAGTTATGTGCACTATTAA
		empA-R	ACAAAGAAGTCGACTAAATAAACCAT
		empA-qF	AAGTCCGTTACCAGCAGATG
		empA-qR	CGTAAACTTGGCCGATACCT
		empA-P	[6-FAM]TCTCAGTTTGCGTTGCTACGACTGAC[BHQ1]
Val	*toxR*	toxR-F	GTGGAACGCTTGAGCCCATT
		toxR-R	GCGTAGTGGGCCGACAGTAT
		gyrB-qF	GTGTACCGGTGATGACACCTG
		gyrB-qR	AATCACTTCAACTGGCAACGAG
		gyrB-P	[HEX]CACGTAGCGCTCAATGCATTGCTC[BHQ1]
Vh	*vhhP2*	vhhP2-F	ATGAAGAGAAGGAATCCTCAAGG
		vhhP2-R	TTATTCCAATCTAGTTGGTTTTGATG
		vhhR2-qF	CATCCTAGCTGTTGTTGCAGCT
		vhhR2-qR	GTTCCACCATTGACTAACCATTGG
		vhhR2-P	[ROX]TGGGTAAATCGCACGCCTGTTTCGA[BHQ2]
Vsc	*luxR*	luxR-F	ATGGACTCTATAGCAAAAAGACCC
		luxR-R	TTACGCTTCTTCTTTGTAAATACACAG
		luxR-qF	GTCGTGGTCATGCCGATATT
		luxR-qR	ATTAGAGAACTGACGTACCACATAGTTC
		luxR-P	[Cy5] CAACTACTTCCCAACACGTGAAGAC[BHQ2]

**Table 2:** Optimal qPCR system.

Component	Volume (μL)
empA-qF(20 μM)	0.3
empA-qR(20 μM)	0.3
empA-qP(20 μM)	0.5
toxR-qF(20 μM)	0.2
toxR-qR(20 μM)	0.2
toxR-qP(20 μM)	0.6
vhhR2-qF(20 μM)	0.4
vhhR2-qR(20 μM)	0.4
vhhR2-qP(20 μM)	0.5
luxR-qF(20 μM)	0.2
luxR-qR(20 μM)	0.2
luxR-qP(20 μM)	0.3
DNA-Va	1
DNA-Val	1
DNA-Vh	1
DNA-Vsc	1
Pro Taq HS Premix Probe real-time PCR Kit III	10
ddH_2_O	Up to 20

**Table 3:** Reproducibility of the multiplex qPCR method.

		Intra-Assay		Inter-Assay	
Targets	Templates (Copies/µL)	Cq Value (Mean ± SD)	CV/%	Cq Value (Mean ± SD)	CV/%
*empA*	10^6^	22.02 ± 0.18	0.82	23.33 ± 0.21	0.90
	10^5^	24.83 ± 0.26	1.05	26.49 ± 0.24	0.91
	10^4^	27.05 ± 0.13	0.48	29.11 ± 0.14	0.48
*toxR*	10^6^	25.12 ± 0.35	1.39	24.99 ± 0.31	1.24
	10^5^	28.22 ± 0.62	1.20	27.73 ± 0.35	1.26
	10^4^	31.00 ± 0.77	1.48	30.46 ± 0.27	0.88
*vhhR2*	10^6^	23.47 ± 0.07	0.30	21.97 ± 0.09	0.41
	10^5^	26.44 ± 0.11	0.42	25.35 ± 0.05	0.20
	10^4^	29.76 ± 0.06	0.20	28.82 ± 0.14	0.49
*luxR*	10^6^	18.04 ± 0.14	0.78	18.85 ± 0.10	0.53
	10^5^	21.41 ± 0.17	0.79	21.71 ± 0.19	0.86
	10^4^	24.54 ± 0.21	0.86	24.59 ± 0.17	0.69

**Table 4:** Analysis of clinical and environmental samples using the multiplex qPCR method.

Different Sample	Sample Size	Test Result (Positive/Negative)	Accuracy Rate/%
**Clinical Sample**			
NC ^a^	9	0/9	100
Va	6	6/0	100
Val	6	6/0	100
Vh	6	6/0	100
Vsc	6	6/0	100
Va+Val	3	3/0	100
Va+Vh	3	3/0	100
Va+Vsc	3	3/0	100
Val+Vh	3	3/0	100
Val+Vsc	3	3/0	100
Vh+Vsc	3	3/0	100
Va+Val+Vh	3	3/0	100
Va+Val+Vsc	3	3/0	100
Val+Vh+Vsc	3	3/0	100
Va+Val+Vh+Vsc	3	3/0	100
**Water Sample**			
NC ^a^	6	0/6	100
Va	3	3/0	100
Val	3	3/0	100
Vh	3	3/0	100
Vsc	3	3/0	100
Va+Val+Vh+Vsc	3	3/0	100
**Sediment Sample**			
NC ^a^	6	0/6	100
Va	3	3/0	100
Val	3	3/0	100
Vh	3	3/0	100
Vsc	3	3/0	100
Va+Val+Vh+Vsc	3	3/0	100
**Positive Control**			
pVa	3	3/0	100
pVal	3	3/0	100
pVh	3	3/0	100
pVsc	3	3/0	100

^a^ Samples from the fish injected with PBS.

## Data Availability

The data presented in this study are available upon request from the corresponding author.

## Supplementary Materials

The following supporting information can be downloaded at: https://www.mdpi.com/article/10.3390/vetsci12040327/s1, Figure S1: Construction of standard plasmids; Table S1: Bacterial strains used for multiple sequence alignment.; Table S2: Cq values of the clinical samples; Table S3: Cq values of the environmental samples.
