# Supplementary material for: Development of Multiple Real-Time Fluorescent Quantitative PCR for Vibrio Pathogen Detection in Aquaculture
Source: Vet Sci. 2025 Apr 2;12(4):327. doi: 10.3390/vetsci12040327 (PMC12030866; doi:10.3390/vetsci12040327)

Supplementary data

Table S1 Bacterial strains used for multiple sequence alignment.

Target gene	Bacterial strain	Accession number	Target gene	Bacterial strain	Accession number
empA	*Vibrio anguillarum empA*	L02528.1	*toxR*	*Vibrio alginolyticus toxR*	KJ579443.1
	Vibrio anguillarum J382	CP091186.1		*Vibrio alginolyticus* HY9901	KJ579443.1
	Listonella anguillarum	AY046320.1		*Vibrio alginolyticus* ZLV3	CP082315.1
	Vibrio anguillarum ATCC-68554	CP023209.1		*Vibrio alginolyticus* Vb1833	CP060386.1
	Vibrio anguillarum MHK3	CP022469.1		*Vibrio alginolyticus* FDAARGOS_114	CP014045.1
	Vibrio anguillarum PF7	CP011465.1		*Vibrio alginolyticus* a10-6-23	AB372529.1
	Vibrio aestuarianus 08_114_4T3	CP091996.1		*Vibrio alginolyticus* B1BNVF9	LR860673.1
	Vibrio proteolyticus	M64809.1		*Vibrio rotiferianus* LPD 1-1-84	FM202708.1
	Vibrio navarrensis	CP051120.1		*Vibrio rotiferianus* H10	FR719008.1
	Vibrio hyugaensis 090810a	CP025795.1		*Vibrio rotiferianus* B1CBC12	LR860721.1
	Shewanella bicestrii JAB-1	CP022358.1		*Photobacterium damselae*	KU760757.1
	Shewanella putrefaciens SA70	CP078038.1		*Photobacterium damselae subsp. damselae* 64bp	KU760751.1
	Pseudoalteromonas carrageenovora KCTC 22325	CP027524.1		*Photobacterium damselae subsp. damselae* 158dp	KU760748.1
	Aeromonas hydrophila vAh ST251	LR963132.1		*Vibrio anguillarum*	KU360395.1
	Aeromonas hydrophila NN-MR659	CP124746.1		*Vibrio harveyi vhhP2*	AY247418.1
toxR	*Vibro scophthalmi luxR*	JN684210.1		*Vibrio harveyi* CAIM 512	DQ503438.1
	Vibro scophthalmi CECT 5965	AFA36062.1		*Vibrio harveyi* VIB 653	DQ640260.1
	Vibro scophthalmi LMG 19158	AFA36061.1	*vhhP2*	*Vibrio harveyi vhhP2*	FJ025787.1
	Vibrio alginolyticus BCT	ABU88077.1		*Vibrio harveyi* 2011V-1164	CP035693.1
	Vibrio campbellii isolate BF5_0283	OX411212.1		*Vibrio harveyi* A2	CP118530.1
	Vibrio owensii V180403	CP033144.1		*Vibrio harveyi* 160-19	CP118532.1
	Vibrio harveyi NH-LM1	CP150467.1		*Vibrio harveyi* WXL538	CP045070.1
	Vibrio rotiferianus B64D1	CP018312.1		*Vibrio owensii* v2	CP166907.1
	Vibrio alginolyticus XWV9	CP082319.1		*Vibrio owensii* V180403	CP033144.1
	Vibrio diabolicus strain NV27	CP085843.1		*Vibrio owensii* SH14	CP045859.1
				Vibrio owensii 1700302	CP033137.1

Table S2 Cq values of the clinical samples.

Sample	Cq value (mean±SD)	Sample	Cq value (mean±SD)	Sample	Cq value (mean±SD)	Sample	Cq value (mean±SD)
NC12-1	37.8±0.5	Val24-1	23.3±0.1	Va+Vsc-24	25.3±0.1	Va+Val+Vsc-12	32.4±0.2
NC12-2	36.4±0.3	Val24-2	26.4±0.3		27.7±0.3		31.2±0.3
NC12-3	NA	Val48-1	21.1±0.2	Va+Vsc-48	20.8±0.3		33.1±0.2
NC24-1	NA	Val48-2	19.7±0.3		23.5±0.2	Va+Val+Vsc-24	25.3±0.1
NC24-2	35.7±0.4	Vh12-1	30.4±0.2	Val+Vh-12	32.2±0.2		23.3±0.3
NC24-3	36.2±0.2	Vh12-2	28.7±0.1		33.2±0.4		26.2±0.1
NC48-1	NA	Vh24-1	23.5±0.1	Val+Vh-24	27.3±0.2	Va+Val+Vsc-48	22.4±0.2
NC48-2	NA	Vh24-2	22.1±0.3		26.8±0.1		24.5±0.1
NC48-3	38.1±0.1	Vh48-1	18.2±0.5	Val+Vh-48	21.7±0.3		25.2±0.2
pVa1	28.1±0.1	Vh48-2	20.7±0.2		20.2±0.2	Val+Vh+Vsc-12	33.1±0.3
pVa2	25.7±0.2	Vsc12-1	33.2±0.1	Val+Vsc-12	31.5±0.2		29.8±0.1
pVa3	22.0±0.4	Vsc12-2	34.1±0.1		33.3±0.2		31.9±0.2
pVal1	27.3±0.3	Vsc24-1	29.1±0.3	Val+Vsc-24	25.1±0.4	Val+Vh+Vsc-24	27.4±0.3
pVal2	23.9±0.1	Vsc24-2	30.3±0.2		27.7±0.4		25.3±0.1
pVal3	20.1±0.4	Vsc48-1	26.7±0.1	Val+Vsc-48	23.4±0.3		26.5±0.2
pVh1	27.9±0.3	Vsc48-2	23.9±0.2		24.1±0.2	Val+Vh+Vsc-48	24.3±0.1
pVh2	24.1±0.5	Va+Val-12	31.3±0.2	Vh+Vsc-12	29.5±0.1		21.4±0.2
pVh3	21.5±0.2		33.2±0.2		32.1±0.4		23.3±0.2
pVsc1	28.1±0.2	Va+Val-24	26.7±0.1	Vh+Vsc-24	23.2±0.3	Va+Val+Vh+Vsc-12	32.1±0.1
pVsc2	25.6±0.1		23.4±0.3		26.7±0.1		31.7±0.2
pVsc3	22.3±0.2	Va+Val-48	21.4±0.3		20.1±0.2		30.8±0.2
Va12-1	30.9±0.2		20.2±0.2	Vh+Vsc-48	24.3±0.2		33.2±0.1
Va12-2	32.6±0.3	Va+Vh-12	32.2±0.1	Va+Val+Vh-12	30.3±0.3	Va+Val+Vh+Vsc-24	32.7±0.2
Va24-1	25.3±0.2		30.6±0.3		32.5±0.2		26.3±0.3
Va24-2	22.5±0.1	Va+Vh-24	27.3±0.2		30.1±0.4		26.9±0.3
Va48-1	18.4±0.1		24.1±0.1	Va+Val+Vh-24	27.3±0.1		25.3±0.2
Va48-2	20.7±0.3	Va+Vh-48	22.5±0.4		25.9±0.3	Va+Val+Vh+Vsc-48	27.2±0.2
Val12-1	33.4±0.1		19.9±0.1		25.1±0.3		24.3±0.4
Val12-2	31.4±0.4	Va+Vsc-12	33.1±0.2	Va+Val+Vh-48	24.4±0.2		22.3±0.2
			32.7±0.3		23.1±0.1		21.4±0.2
					22.3±0.3		24.2±0.3

Table S3 Cq values of the environmental samples.

Water sample	Cq value (mean±SD)	Sediment sample	Cq value (mean±SD)	Positive sample	Cq value (mean±SD)
NC1	NA	NC1	38.7±0.2	pVa1	28.4±0.2
NC2	NA	NC2	37.5±0.3	pVa2	25.3±0.1
NC3	NA	NC3	NA	pVa3	22.4±0.2
NC4	NA	NC4	NA	pVal1	27.1±0.2
NC5	NA	NC5	38.7±0.1	pVal2	23.4±0.2
NC6	NA	NC6	NA	pVal3	20.5±0.2
Va1	30.2±0.3	Va1	31.7±0.1	pVh1	27.5±0.2
Va2	26.5±0.2	Va2	27.8±0.3	pVh2	24.4±0.2
Va3	23.2±0.7	Va3	25.7±0.1	pVh3	21.1±0.3
Val1	28.3±0.2	Val1	30.1±0.3	pVsc1	28.5±0.1
Val2	25.2±0.6	Val2	27.8±0.3	pVsc2	25.2±0.2
Val3	23.8±0.3	Val3	24.3±0.1	pVsc3	22.7±0.1
Vh1	28.5±0.4	Vh1	29.4±0.2		
Vh2	24.1±0.4	Vh2	25.9±0.4		
Vh3	22.1±0.2	Vh3	22.4±0.1		
Vsc1	32.3±0.4	Vsc1	30.5±0.3		
Vsc2	27.1±0.2	Vsc2	26.8±0.1		
Vsc3	24.5±0.2	Vsc3	24.1±0.2		
Va+Val+Vh+Vsc-1	29.2±0.2	Va+Val+Vh+Vsc-1	27.4±0.1		
	28.2±0.1		29.1±0.2		
	27.4±0.3		28.6±0.2		
	28.7±0.1		28.3±0.3		
Va+Val+Vh+Vsc-2	22.8±0.2	Va+Val+Vh+Vsc-2	23.1±0.3		
	22.6±0.2		21.9±0.1		
	24.5±0.3		23.1±0.4		
	24.5±0.2		22.6±0.3		
Va+Val+Vh+Vsc-3	29.1±0.1	Va+Val+Vh+Vsc-3	25.3±0.1		
	24.5±0.1		29.1±0.1		
	27.6±0.3		28.3±0.2		
	25.3±0.2		24.1±0.1		

Figure S1 Construction of standard plasmids. Standard plasmids pempA (A), ptoxR(B), pVhhR2 (C), and pluxR (D) recombined with *empA*, *toxR*, *VhhR2*, and *luxR* genes, respectively, were validated with conventional PCR.

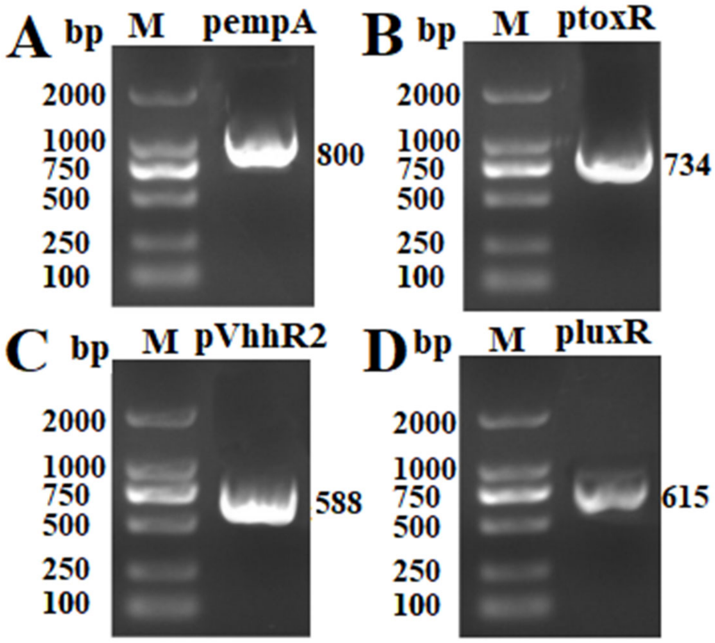

Supplement: Supplementary file 1 [file vetsci-12-00327-s001.zip › vetsci-3485096-supplementary.pdf]
